# The Poor Man's Home.—XXII

**Published:** 1898-01-22

**Authors:** 


### Modern Sociology.

### THE POOR MAN'S HOME.—XXII.

### Patching up Old Property.

Hitherto we have spoken only of new buildings, erected especially for the working classes, and adapted as well as might be to their tastes and needs. Such are almost invariably the best; but sanitary science and its application to the dwellings of the poor is such a new thing that there are in every part of the country houses, not always dilapidated nor even uncomfortable, which are nevertheless unhealthy. To destroy all these and build new ones, according to the last word of sanitation, is too costly a thing to be contemplated. Many who, as ratepayers, would be called on to pay for such improvements, themselves live in houses which leave a good deal to be desired, and we may be sure that they will refuse to sanction any great expenditure on the destruction of houses which are not to the ordinary eye unfit for human habitation. While we may wish that all houses which are not thoroughly satisfactory from a sanitary point of view might be got rid of, it is as well to admit that we might as well demand that no one in these islands should ever go with a patch upon his clothing. Patching there must be; we hope to show how such patching should be done.

Houses which have been built for the class that now occupies them may look very dilapidated, and may be for the moment unfit for habitation, without being by any means hopeless. If the brick or stonework of the walls still holds the house can be reconstructed, though sometimes at an expense which is not much smaller than the cost of building a new one. As it is almost certain that the drainage will be defective attention must first be paid to it. It is usually best to shut up old drains altogether unless their course can be traced with absolute certainty. The plumber of bygone days was neither a very intelligent nor a very honest man, and cases have been known where he satisfied the owner of the property by leading a drain pipe into the ground and cutting it short below the surface, leaving the sewage to soak as it would through the soil beneath the house. Where there is any suspicion of such practices having been followed, the only course is to shut up the suspected drain, and make a new one. Where the closets and sinks are inside the houses, it is generally best to remove them to the outside. The class who occupy these patched-up dwellings are, as a rule, the poorest of the working class, the labouring people, who are often ignorant and unintelligent. They know nothing of what a drain will, and what it will not, take away; everything that will not go down the sink is put down the closet, and if by pulling a plug they can get it out of sight, they are perfectly satisfied. Drains thus get choked with filth, and it is only when the pipe will contain no more that it even occurs to them that anything is wrong. Indeed, for that class, the old-fashioned privy, which, as we have seen, still obtains on the Continent, is really more healthy than the apparently superior concealed drain, flushed more or less satisfactorily. It can be made an ashpit of without danger to health, and, being so perceptibly offensive, it has a better chance of being cleaned at frequent intervals. It does not pretend to carry off the matters thrown into it, and thus no one being deceived there is a better chance of its being kept in proper order.

The other defects most common in old property are dilapidated woodwork, gaps in the lead gutters of the roof, and deficiencies in the pointing of the walls. Wooden stairs should, as a rule, be entirely renewed. To put a new bit of wood only where the old step is worn is but poor economy. The old wood breaks away from the new, leaving a hole and an outstanding piece, which is apt to trip up anyone who goes up or down, and the carpenter will be in endless requisition. It is wiser to replace wooden balusters with iron ones, as the wooden ones form too tempting a substitute for firewood. As the skirting round the walls is almost certain to have shrunk, it is well to fill up the space between it and the walls with putty. It is hardly worth while to put in new doors or shutters because the previous tenant has expressed his artistic or amorous soul by carving them with a pocket-knife; the incoming tenant or his sons will probably do the same. Only if doors and window-sashes are too loose, and let in a draught, is it needful to meddle with them. It is so obviously to the landlord's interest to keep the pointing of the house good, that it would be unnecessary to speak of it, if it were not that so many landlords neglect it. The same may be said of the lead work. Any faults in the gutters that go round the roof, and in the pipes that lead from it, are sure to make the walls damp, and therefore injure the whole property. Repairs of this sort, if properly carried out, will often make a good and—which must not be ignored—profitable possession of a house which would soon have become uninhabitable.

When the building itself is fundamentally at fault the matter is more difficult. This is the case with what are called "back-to-back" houses. In these there is no through ventilation, and even when they are, so far as their occupants know, good enough, they are inevitably unhealthy. Where, as we have seen at Mulhouse, the house has two sides exposed to the open air, although there is no through ventilation, it may be tolerably healthy; but a back-to-back house in a row is hopeless as a sanitary dwelling. Two plans are possible for remedying the fundamental faults of such a house. Either the two houses which stand back to back may be thrown into one, or every third pair of houses may be destroyed. The former method has the disadvantage of converting two small and, perhaps, convenient houses into one large and probably ill-arranged one. The mere driving of a passage through between the two houses does not, indeed, make a house healthy, though it may create a draught; and the rearrangement of rooms which would make the most of the area under the new conditions is a thing too expensive to be often properly carried out. The other plan, which cuts up the row into blocks, each containing four houses, after the Mulhouse example, is much better, as it leaves the original arrangement of each tenement, which is often in no way bad. It is, however, attended with considerable loss, as a third of the rental of the street must be sacrificed by the pulling down of the sets of houses at the fixed intervals.

